# ﻿A new species of *Hiptage* (Malpighiaceae) from northwest Yunnan (China) based on molecular and morphological data

**DOI:** 10.3897/phytokeys.232.106675

**Published:** 2023-09-04

**Authors:** Tong-Tong Zhang, Shu-Yun Yang, Ke Tan, Ming-Xun Ren

**Affiliations:** 1 Key Laboratory of Genetics and Germplasm Innovation of Tropical Special Forest Trees and Ornamental Plants, Ministry of Education, Hainan University, Haikou 570228, China Hainan University Haikou China; 2 Guangxi Key Laboratory of Plant Conservation and Restoration Ecology in Karst Terrain, Guangxi Institute of Botany, Guangxi Zhuang Autonomous Region and Chinese Academy of Sciences, Guilin 541006, Guangxi, China Guangxi Institute of Botany Guilin China

**Keywords:** flora of Yunnan, Hengduan Mountains, *Hiptage*, Malpighiaceae, taxonomy

## Abstract

*Hiptagestenopterum* K.Tan & M.X.Ren, a new species of *Hiptage* collected from a deep valley close to the Nujiang Gorge, northwest of Yunnan Province, China, is described and illustrated based on molecular and morphological data. The new species was found isolated in an entrenched valley of the Laowo River, a tributary of the Nujiang River, at the northern edge of the distribution range of the genus. *H.stenopterum* shares some morphological similarities with the narrowly endemic *H.incurvatum* and *H.lushuiensis*. However, *H.stenopterum* is easily distinguished by its oblanceolate lateral wing of winged mericarp, 10 to 12 calyx glands, and branchlets densely rusty tomentose. The new species status is also supported by molecular phylogenetic analyses based on nuclear ribosome internal transcribed spacer (nrITS), which showed distinct systematic boundaries from the most morphologically similar species, *H.incurvatum* and their morphological relatives, *H.lushuiensis*.

## ﻿Introduction

*Hiptage* Gaertn. (Gaertner 1791) is a large genus of Malpighiaceae, currently comprising ca. 40 species of woody shrubs or lianas growing at shrub forests or valleys on limestone hills or riverbanks of tropical Asia, such as Indo-China Peninsular, Malay Archipelago and Southern China (Sirirugsa 1991; Hô 1992; Srivastava 1992; Chen and Funston 2008; Ren 2015; Yang et al. 2018; Tan et al. 2019; Dong et al. 2020; Wei et al. 2022). *Hiptage* is derived from the Greek hiptamai, which means “to fly” and refers to its unique three-winged fruit. Such three-winged fruit means it is easily dispersed over long distances, explaining its widespread distribution in tropical Asia (Sirirugsa 1991; Srivastava 1992; Hô 1992; Chen and Funston 2008). Nonetheless, most species of this genus are narrow and endangered endemics, especially in the ravine and mountain peaks, such as *H.multiflora* F.N.Wei, *H.lushuiensis* S.P.Dong, K.Tan & M.X.Ren, and *H.incurvatum* K. Tan & M.X. Ren (Wei 2018; Tan et al. 2019; Dong et al. 2020). In recent years, five new species of *Hiptage* have been described. One species was found in Southern India (Sujana and Vadhyar 2020), and four were collected in Southwestern China (Yang et al. 2018; Tan et al. 2019; Dong et al. 2020).

*Hiptage* is currently classified within the Tetrapteroid clade, which is among the ten primary lineages identified for Malpighiaceae (Davis and Anderson 2010). Within that phylogenetic framework, *Hiptage* included six species and was resolved as closely related to *Flabellariopsis* R.Wilczek, a genus endemic to Africa. *Hiptage* and *Flabellariopsis* comprise a clade with weak support, sister to the Neotropical genus *Carolus* W.R.Anderson (Davis and Anderson 2010). Despite relying on a single marker (ITS) and yielding mainly weakly supported clades, recent molecular phylogenies of *Hiptage* indicate that *H.stellulifera* Arènes stands as the basal group within the genus (Tan et al. 2019; Almeida and van den Berg 2022).

During our fieldwork in the Hengduan Mountains, northwest of Yunnan Province, China, we collected specimens from a population of a distinct morphotype of *Hiptage* growing on a hillside in the valley of Laowo River, close to the Nujiang Gorge. After detailed analyses of morphological characters and molecular data of individuals from this population, we found out that this population is most morphologically similar to *H.incurvatum* and *H.lushuiensis*. But it differs in the number of calyx glands and the morphology of mericarp wings, both of which are critical morphological traits for species taxonomy in *Hiptage* (Chen and Funston 2008; Ren 2015). Therefore, we describe it as a new species, depicted and illustrated here.

## ﻿Materials and methods

### ﻿Taxonomy

Measurements and observations of morphological characters of the new species were based on fresh and herbarium specimens. Each character was measured across five individuals. Photographs of fresh plants and floral characteristics were taken in the field. The new species was compared with all type specimens of *Hiptage* in morphology from protologues and specimens in the herbarium (at the herbaria IBK, KUN, and IBSC). Images of type specimens of all *Hiptage* species were consulted from JSTOR Global Plants (http://plants.jstor.org), the Chinese Virtual Herbarium (http://www.cvh.ac.cn), and taxonomic literature (e.g., Yang et al. 2018; Tan et al. 2019; Dong et al. 2020). The morphological terminology follows Niedenzu (1924), Jacobs (1955), Anderson et al. (2006), Chen and Funston (2008), Ren (2015), and Almeida and Morais (2022). The conservation status assessments of the new species *H.stenopterum* were based on the International Union for Conservation of Nature guidelines (IUCN 2022).

### ﻿Phylogenetic analysis

To verify the taxonomic status of the new species within *Hiptage*, we conducted a phylogenetic analysis using the nuclear ribosomal internal transcribed spacer (ITS) region. We downloaded ITS sequence data for 42 samples from GenBank (Table 1) encompassing 16 species of *Hiptage* and, based on the study of Almeida and van den Berg (2022), four species from the Tetrapteroid clade serving as outgroups [i.e. *Callaeumpsilophyllum* (A.Juss.) D.M.Johnson, *Heteropterysbrunnea* R.Sebast. & Mamede, *Niedenzuellamultiglandulosa* (A.Juss.) W.R.Anderson and *N.stannea* (Griseb.) W.R.Anderson] (Table 1). Dried leaf material of the proposed new species was collected from the type locality in a valley of the Laowo River, close to Lushui City (Yunnan, China). Five samples from the new species and three samples from *H.lushuiensis* were newly sequenced in this study to provide molecular evidence (Table 1). Total genomic DNA was isolated from dried leaf samples using a CTAB protocol adapted from Doyle and Doyle (1987). PCR amplification, in 25 μl reactions, comprised 1 μl of sample DNA, 12.5 μl of 2× Taq PCR Master Mix (Aidlab Biotechnologies Co. Ltd), 1μl of each primer (10 μmol/ml), with double distilled water making up the remainder. Amplification of the ITS region utilized primers ITS17SE and ITS26SE as per Sun et al. (1994). The amplification regime involved a 5 min initial denaturation at 94 °C, then 35 cycles of 40s at 94 °C, 20s at 69 °C, 1 min at 72 °C, and concluded with a 10 min extension at 72 °C. The resulting PCR products were bidirectionally sequenced on an ABI3730XL platform.

**Table 1.** Taxa, GenBank accession numbers, voucher numbers of *Hiptage* used in this study.

Species	Locality	GenBank Accession	Voucher Number
*Hiptagebenghalensis* (L.) Kurz	Yangjie, Yunnan, China	MH718400	M. X. Ren & L. Tang 128 (HUTB)
	Menglian County, Yunnan, China	MH718422	S. P. Dong 131 (HUTB)
	Daxin County, Guangxi, China	MH718414	K. Tan & S. P. Dong 95 (HUTB)
	Lekang County, Guizhou, China	MH718415	K. Tan, S. P. Dong, & M. X. Ren 82 (HUTB)
	Singapore	MH718399	T. W. Yam 3334 (HUTB)
	Phatthaya, Thailand	MH718408	K. Tan, S. P. Dong, & M. X. Ren 3344 (HUTB)
	Chiangmai, Thailand	MH718410	K. Tan, S. P. Dong, & M. X. Ren 3336 (HUTB)
*H.multiflora* F.N.Wei	Nonggang National Nature Reserve, Guangxi, China	MH718424	K. Tan & S. P. Dong 52 (HUTB)
*H.stenopterum* K. Tan & M.X. Ren	Lushui City, Yunnan, China	OQ968812	tank 190402001 (HUTB)
		OQ968813	–
		OR417356	23tk041401(IBK)
		OR417357	23tk041402(IBK)
		OR417358	23tk041403(IBK)
*H.incurvatum* K. Tan & M.X. Ren	Pingpo Town, Yunnan, China	MK967956	K. Tan, H. L. Zheng, & M. X. Ren 201903309 (HUTB)
		MK967957	K. Tan, H. L. Zheng, & M. X. Ren 201903310 (HUTB)
		MK967958	K. Tan, H. L. Zheng, & M. X. Ren 201903305 (HUTB)
		MK967959	K. Tan, H. L. Zheng, & M. X. Ren 201903306 (HUTB)
*H.monopteryx* Sirirugsa	Phatthaya, Thailand	MH718407	K. Tan, S. P. Dong, & M. X. Ren 3337 (HUTB)
*H.marginata* Arènes	Hue, Vietnam	MH718413	K. Tan & Q. Yang 3363 (HUTB)
*H.detergens* Craib	KuiBuri, Thailand	MH718404	K. Tan, S. P. Dong, & M. X. Ren 3328 (HUTB)
	Sam Roi Yot, Thailand	MH718405	K. Tan, S. P. Dong, & M. X. Ren 3326 (HUTB)
*H.lucida* Pierre	Phatthaya, Thailand	MH718406	K. Tan, S. P. Dong, & M. X. Ren 38 (HUTB)
	Xishuangbanna, Yunnan, China	MH718418	Z. N. Qian & S. P. Dong120 (HUTB)
*subglabra* Arènes	Nui Chua National Park, Phan Rang, Vietnam	MH718427	K. Tan & S. J. Ling 3364 (HUTB)
*H.bullata* Craib	Lampang, Thailand	MH718412	K. Tan, S. P. Dong, & M. X. Ren 3320 (HUTB)
*H.minor* Dunn	Lekang County, Guizhou, China	MH718398	K. Tan, S. P. Dong, & M. X. Ren 79 (HUTB)
	Wenshan City, Yunnan, China	MH718423	K. Tan, S. P. Dong, & M. X. Ren 94 (HUTB)
	Lushui City, Yunnan, China	MH718401	K. Tan, S. P. Dong, & M. X. Ren 88 (HUTB)
*H.ferruginea* Y.H.Tan & Bin Yang	Xishuangbanna, Yunnan, China	MH718402	S. P. Dong 116 (HUTB)
	Xishuangbanna, Yunnan, China	MH718403	S. P. Dong 117 (HUTB)
*H.pauciflora* Y.H.Tan & Bin Yang	Menglian County, Yunnan, China	MH718420	S. P. Dong 73 (HUTB)
*H.umbellulifera* Arènes	Cana, Phan Rang, Vietnam	MH718426	K. Tan & S. J. Ling 3386 (HUTB)
	Phan Rang, Vietnam	MH718430	K. Tan & S. J. Ling 3399 (HUTB)
	Nui Chua National Park, Phan Rang, Vietnam	MH718428	K. Tan & S. J. Ling 3385 (HUTB)
*H.luzonica* Merr.	Luzon Island, Philippines	MH718425	K. Tan, W. Q. Xiang & M. X. Ren 20191181436 (HUTB)
	Palawan Island, Philippines	MH718432	K. Tan, W. Q. Xiang & M. X. Ren 3305 (HUTB)
	Cebu Island, Philippines	MH718431	K. Tan & W. Q. Xiang 3301(HUTB)
*H.candicans* Hook.	Chiangmai, Thailand	MH718409	K. Tan, S. P. Dong, & M. X. Ren 3328 (HUTB)
	Chomthong, Thailand	MH718411	K. Tan, S. P. Dong, & M. X. Ren 3330 (HUTB)
*H.stellulifera* Arènes	NhaTrang, Vietnam	MH718429	K. Tan & S. J. Ling 3376 (HUTB)
*H.lushuiensis* S.P.Dong, K.Tan & M.X.Ren	Lushui City, Yunnan, China	OR471605	S. P. Dong 176 (HUTB)
		OR471606	S. P. Dong 177 (HUTB)
		OR471607	S. P. Dong 178 (HUTB)
*Heteropterysbrunnea* R.Sebast. & Mamede	–	OK284366	RFAlmeida 579 (HUEFS)
*Callaeumpsilophyllum* (A.Juss.) D.M.Johnson	–	OK268022	RFAlmeida 734 (HUEFS)
*Niedenzuellamultiglandulosa* (A.Juss.) W.R.Anderson	–	OK271417	RFAlmeida 639 (HUEFS)
*Niedenzuellastannea* (Griseb.) W.R.Anderson	–	OK271412	Pott 1816 (HUEFS)

The sequencing results of ITS fragments were evaluated with PhyDE (Müller et al. 2010) for base confirmation and contiguous sequence editing. All sequences were manually aligned in MEGA v.7 (Kumar et al. 2016). Some erroneous sequencing results were excluded from the alignment range. The Bayesian inference (BI) and Maximum likelihood (ML) analyses were used in Phylosuite (Zhang et al. 2020) to construct the phylogenetic tree of *Hiptage*. The SYM+G4 model and the K2P+G4 model as the best-fit substitution model for ML and BI analysis, respectively, using ModelFinder (Kalyaanamoorthy et al. 2017) with Corrected Akaike Information Criterion (AICc). Bayesian Inference started with a random tree as a simulated tree, used a Markov chain Monte Carlo (MCMC) to run simulations for 10 million generations and sampled every 1000 generations, and was performed using MrBayes v3.2.5 (Ronquist et al. 2012). The first 2500 trees (25% of the total trees) were discarded as burn-in samples. The maximum likelihood (ML) analyses were accomplished with IQ-TREE v.2.0.6 (Nguyen et al. 2015) with 1000 bootstrap replicates. The final constructed phylogenetic tree was visualized in FigTree v.1.4.3 (http://tree.bio.ed.ac.uk/software/figtree/).

## ﻿Results

The aligned matrix of ITS sequences consisted of 690 bp, of which 466 sites were identical, 132 (19.1%) were parsimony informative, and 92 parsimony-uninformative variable characters. The phylogenetic analysis showed that *Hiptage* is a monophyletic group (PP/BS=1/100), with *H.stellulifera* (PP/BS=1/100) being the first lineage to diverge, consistent with previous studies (Tan et al. 2019; Almeida and van den Berg 2022). The five samples of the proposed new species, *H.stenopterum*, formed a clade with strong support (PP/BS = 0.79/94) sister to a well-supported subclade alongside *H.incurvatum* (PP/BS=0.90/91) (Fig. 3). The results of molecular phylogenetics align with morphological observations, with the new species closely resembling *H.incurvatum* due to the presence of multiple glands. However, *H.stenopterum* is distinguished by its oblanceolate wings, a more significant number of calyx glands, and branchlets that are densely covered with rusty tomentose. Other features, like pedicels, differentiate the two species, as detailed in Table 2. The new species is also similar to *H.lushuiensis* due to the elliptic leaf blade and lanceolate bracteoles. However, there are significant discrepancies between the morphological and molecular phylogenetic findings (Fig. 2, Table 2). Especially in phylogenetics, *H.lushuiensis* was resolved as sister to *H.minor* (PP/BS = 1/99) (Fig. 2).

**Figure 1.**
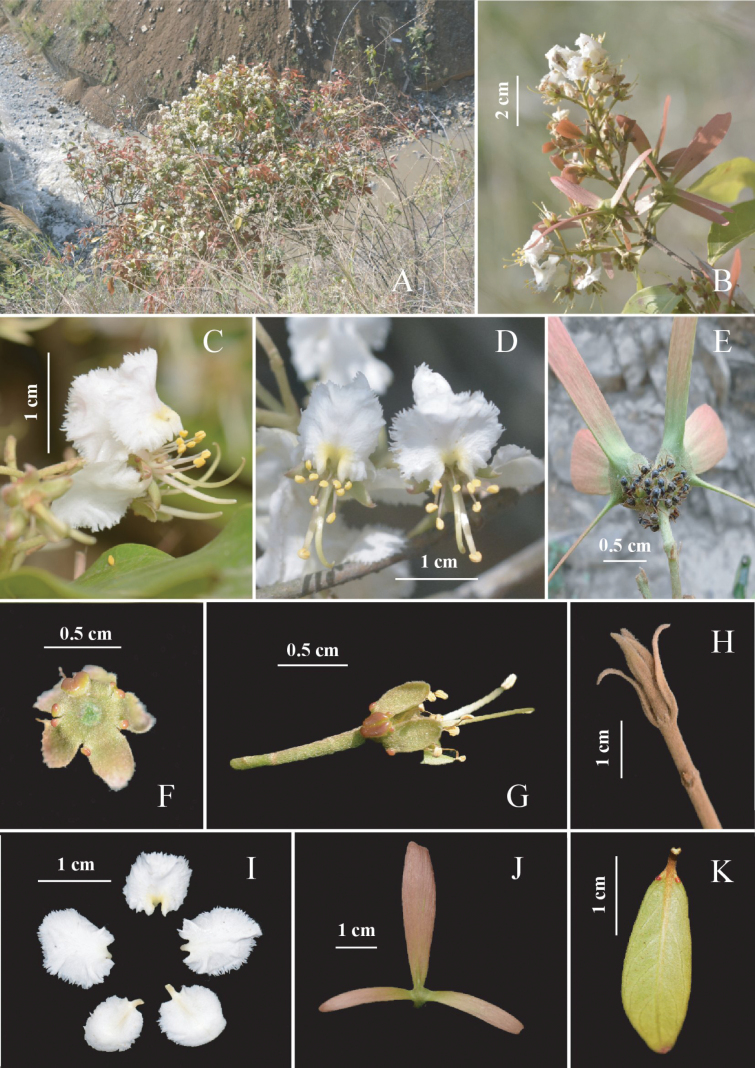
*Hiptagestenopterum***A** habit **B** flowering branch **C** flower in side view **D** flowers in frontal view **E–G** calyx glands (**E** calyx glands with secretions attracting ants) **H** young branch (showing densely rusty tomentose) **I** petals **J** winged mericarp **K** young leaf. Photos **A, B** by M.X. Ren, **E** by T.T. Zhang, and **C, D, F–K** by K. Tan.

**Figure 2.**
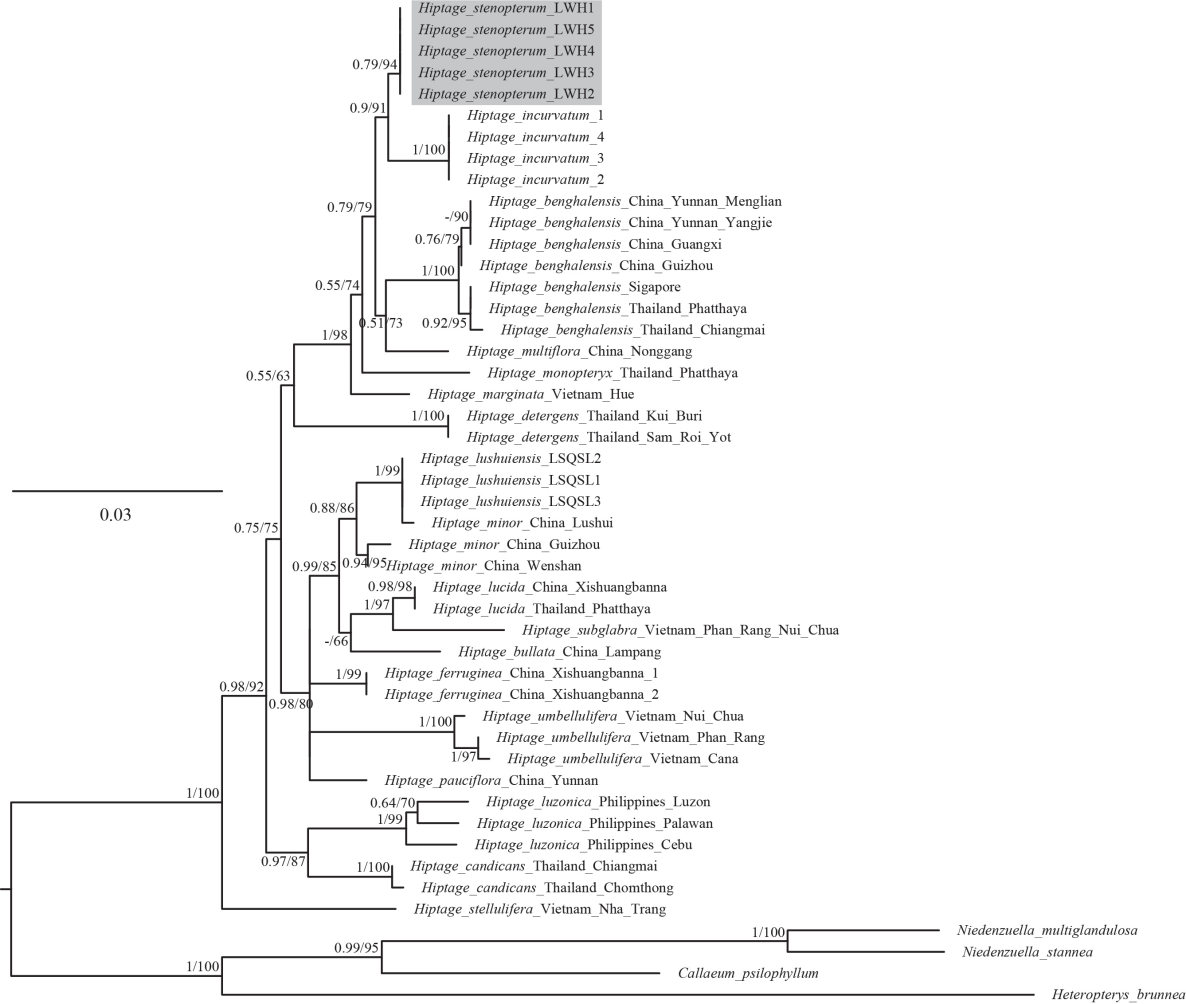
Molecular phylogenetic tree of *Hiptage* based on ITS sequences. Bayesian posterior probability (PP) and ML bootstrap values (BS) are shown above branches as PP/BS (only shown if BS > 50%).

**Figure 3.**
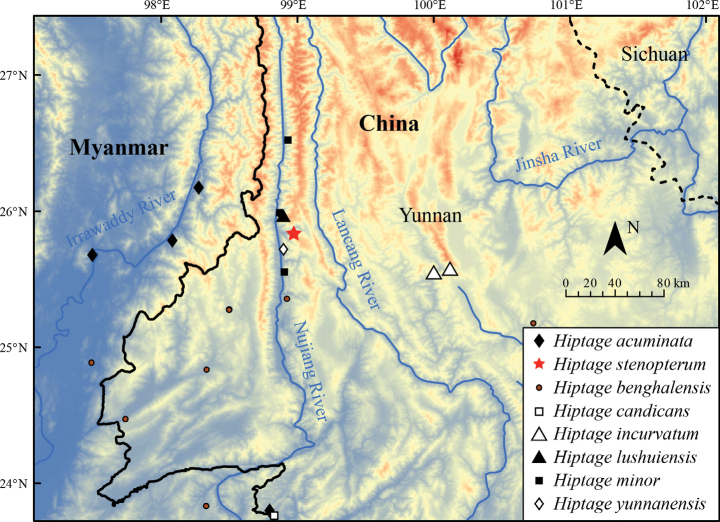
Distribution map showcasing the new species, *Hiptagestenopterum*, alongside the other seven species of the genus found in adjacent regions

**Table 2.** Morphological comparison of key characteristics in *H.stenopterum*, *H.incurvatum*, *H.lushuiensis*, and the most widespread *H.benghalensis*.

Character	*stenopterum*	*H.incurvatum*	*H.lushuiensis*	*H.benghalensis*
Leaf blade	elliptic to oblong, 4.0–12.0 × 2.0–5.0 cm, 2 glands	elliptic, 6–12 × 5–4.5 cm, 10–16 glands	elliptic, 9–16 × 3.5–5.0 cm, 2 or more glands at the base	oblong, elliptic-oblong, or ovate-lanceolate, 9–18 × 3–7 cm, 2 glands
Branchlet	densely rusty tomentose	white to grey sericeous	white to grey sericeous	densely yellowish brown or silver-gray sericeous
Bracteole	lanceolate, ca. 0.5 cm long	lanceolate, 0.3–0.5 cm long	lanceolate, ca. 1.5 mm long	acute, 0.2–0.4 cm long
Pedicel	yellow-brown sericeous, 1.5–2.0 cm long	white sericeous, 1.5–2.5 cm long	white sericeous, ca. 1.5 cm long	sericeous, 0.8–2.5 cm long
Calyx gland	10(-12), two large, ca. 3.0 ×1.0 mm, basally coalescent two smaller adnate to the apex of the two big glands separately, 0.4–0.5 mm diam.; remaining glands 1.8–2.0 × 0.8–1.0 mm, oblong; not decurrent to the pedicel	4-(6), two large, ca. 3.0 × 1 mm, basally connate, remaining glands 0.4–0.5 mm diam., round; not decurrent to the pedicel	2, ca. 1 mm diam., free, sometimes with additional smaller glands on the other sepals, not decurrent along the pedicel	1, very large, oblong, lanceolate to oblanceolate; more or less 1/2 adnate onto the pedicel
Sepal	elliptic to oblong, ca. 0.5 cm long	elliptic to oblong, ca. 0.5 cm long	elliptic, 0.4–0.5 cm long	broadly elliptic or ovate, 0.5–0.6 cm long
Petal	suborbicular, white to light white-pink, 1.0–1.5 × 0.8–1.2 cm	suborbicular, white to light white-pink, ca. 1 × 0.8 cm	suborbicular, pink, ca. 1 cm long	ovate-oblong to suborbicular, white, ca. 1 cm long
Claw	1 mm long, pubescent	1 mm long, pubescent	1 mm long, pubescent	1–2 mm long, glabrous
Posterior lateral wing	oblanceolate, ca. 4.2 × 0.8 cm	ovate, ca. 3.6 ×1.3 cm	ovate, ca. 3.5 × 1.3 cm	oblong, elliptic or obovate-lanceolate, 3–5 × 1–1.6 cm
Anterior lateral wing	lanceolate to linear, straight, ca. 2.5 × 0.4 cm	lanceolate, arcuate back to the middle, ca. 2.3 × 0.7 cm	lanceolate, ca. 2.0 × 0.9 cm	lanceolate-oblong, 1.5–3 cm long

## ﻿Discussion

The zygomorphic, mirror-image, and floral structure of *Hiptage* represents a distinct evolutionary trait of biological significance that promotes adaptability (Ren et al. 2013; Qian et al. 2016). Currently, the presence, shape, and number of calyx glands, as well as their attachment to the pedicel, are critical diagnostic features for species identification in *Hiptage* (Sirirugsa 1991; Srivastava 1992; Chen and Funston 2008; Yang et al. 2018; Tan et al. 2019; Almeida and van den Berg 2022). However, Almeida and van den Berg (2022) contend that the presence and number of calyx glands in *Hiptage* are highly homoplasic and unsuitable for circumscribing infrageneric ranks. Calyx glands in *Hiptage* might be an evolutionary adaptation to attract myrmecophilous interactions for herbivore defense (Ren et al. 2013; Qian et al. 2016). Therefore, the absence and number of calyx glands may be related to local herbivorous insects, which are a significant factor in promoting speciation (McCall and Irwin 2006; Ramos and Schiestl 2019). Based on long-term field observations, we believe that although there is a slight probability of variation in the number of calyx glands, the overall characteristic remains relatively stable. In summary, we believe that the absence and number of calyx glands still hold significance in the infrageneric classification of *Hiptage*. However, it should be properly carried out in future phylogenetic studies to establish a new monophyletic infrageneric classification system.

Morphologically, *H.stenopterum* shares specific characters with *H.incurvatum* and *H.lushuiensis* in having elliptic leaf blades, lanceolate bracteoles, white to light pink flowers, and suborbicular petals. Based on the phylogeny tree, *H.incurvatum* is closely related to the new species, while *H.lushuiensis* clusters with *H.minor*, which has non-reflexed petals. Not only do the petals differ in posture, but there are also significant morphological differences between *H.lushuiensis* and *H.minor* such as the number of calyx and leaf blade glands, and leaf size (Chen and Funston 2008; Dong et al. 2020). Their clustering together might be due to the limited genetic information from just the ITS region and more comprehensive molecular data will be needed in the future to explain this curious relationship.

Malpighiaceae is characterized by an unparalleled diversity of winged fruits in angiosperms (Anderson et al. 2006; Manchester and O’Leary 2010; Tan et al. 2018). Winged mericarps are an adaption for wind dispersal of fruits (Tan et al. 2018), suggesting that such morphological adaptations have significantly facilitated long-distance dispersal and speciation (Davis et al. 2001, 2002; Tan et al. 2018, 2019). Notably, the wings of *H.stenopterum* distinctly deviate from its phylogenetically proximate taxa, *H.incurvatum*. Moreover, these two taxa are partitioned by the Hengduan Mountains, implying that differing aerodynamic conditions, perhaps governed by localized wind patterns, might have driven morphological divergence. Furthermore, the type localities of *H.stenopterum* and *H.incurvatum*, encircled by high mountains and deep gorges, constitute a distinct isolated habitat, which restricts gene flow and facilitates speciation.

### ﻿Taxonomy

#### ﻿ Hiptage stenopterum

Taxon classificationPlantaeMalpighialesMalpighiaceae

K.Tan & M.X.Ren sp. nov.

0E835970-40CC-5A45-A3C7-031033A12D89

urn:lsid:ipni.org:names:77326071-1

Fig. 1

##### Diagnosis.

*Hiptagestenopterum* is most similar to *H.incurvatum* K.Tan & M.X.Ren by branchlets densely rusty tomentose (*vs* white to grey sericeous), leaf blades with 2 glands near the base (*vs* 10–16 glands), 10(-12) calyx glands [*vs* 4 (-6)], the posterior lateral wing oblanceolate (*vs* ovate), anterior lateral wings straight, lanceolate to linear (*vs* arcuate back to the middle, lanceolate).

##### Type.

China. Yunnan Province: Lushui City, Laowo River, 25°50′08″N, 98°54′28″E, 1071 m alt., 2 Apr. 2019, *K. Tan 190402001* (Holotype: HUTB!; Isotype: HUTB!, IBK00450922!).

##### Description.

Woody shrubs; young branches densely rusty tomentose, hairs adpressed, older twigs glabrous, with white or greenish lenticels, rounded, coarse warts dotted. ***Leaves*** opposite; stipules absent; petiole ca. 0.5 cm long, round, tomentose, with yellowish brown hairs, eglandular; leaf blades 4.0–12.0 × 2.0–5.0 cm, coriaceous, elliptic to oblong; young leaves densely rusty tomentose on both surfaces; mature leaves green, glabrous, base obtuse or broadly cuneate, margin plane, apex acuminate, abaxially often with 2 marginal glands near the base; lateral veins in 5–8 pairs, both surfaces prominently. ***Thyrses***, terminal or axillary; main axis 4.0–13.0 cm long, rusty tomentose; peduncle 1.0–2.5 cm, rusty sericeous; bracteoles ca. 0.5 cm long, lanceolate. ***Flowers*** white to slightly pink; pedicels 1.5–2.0 cm long, densely rusty tomentose; sepals 5, 4.5–5.5 × 1.5–2.5 mm, elliptic to oblong, apex obtuse, margin slightly revolute, abaxially densely rusty tomentose, adaxially glabrous. ***Calyx glands*** 10(–12), prominent, not decurrent to the pedicel, often 1 pair of glands at base abaxially; two large, 2.3–2.8 × 0.8–1.2 mm, connate at the base, two smaller glands, 0.4–0.5 diam., rounded, adnate to the apex of the two larger glands separately; remaining glands small and free, 0.5–0.6 × 0.8–1.0 mm, oblong, attached to the margins of other sepals, occasionally one or two glands lacking. ***Petals*** 5, 1.0–1.5 × 0.8–1.2 cm, white to light white to pink, basally yellow, extremely reflexed, suborbicular, margin ciliate, claws ca. 1 mm long, base subcordate to rounded, apex roundish, abaxially densely white tomentose, adaxially glabrous. ***Stamens*** 10, basally fused or free, glabrous, differing in size, pollen sacs rimose; one larger, filament 10–12 mm long, yellowish green, circinate, anther oblong, 1.8–2.0 × 0.7–1.0 mm; 9 smaller stamens, filament 4–6 mm long; anthers oblong, 1–1.3 × 0.7–0.9 mm. ***Ovary*** ca. 2 mm in diam., ovoid, white to rusty tomentose; style 1, yellowish green, 10–13 mm long, slightly curved upwards, deflected either to the left or right side, glabrous; stigma apical. ***Mericarps*** 3, wings pink with yellow-green base, rusty sericeous, posterior lateral wing 3.8–4.5 × 0.7–0.9 cm, oblanceolate, apex roundish or lobed slightly, base obtuse, anterior lateral wings 2.4–3.0 × 0.3–0.6 cm, lanceolate to linear; areole ca. 4–6 mm, approximately triangular. ***Seeds*** angular–globose, 3–5 mm, dark yellow or brown.

##### Phenology.

Flowering in March and fruiting from March to May.

##### Etymology.

Its specific epithet reflects the long and narrow mericarp wings of *Hiptagestenopterum*.

##### Vernacular name.

Chinese: 狭翅风筝果(xiá chì fēng zhēng guǒ). The name’ xiá chì’ means its long and narrow wings, and ‘fēng zhēng gǔo’ is the Chinese name of *Hiptage*.

##### Habitat and distribution.

*H.stenopterum* is only known from a valley of the Laowo River, a tributary of the Nujiang River, at an elevation ca. 1,000 m, near Lushui City, northwest of Yunnan Province, China (Fig. 2).

##### Conservation status.

Since the only known population of *H.stenopterum* is in an entrenched valley of Nujiang River in the northwest Yunnan Province, we have not discovered the wild population outside of the abovementioned place, information known about the population status and natural distribution range of the new species is very limited. Currently, only about 20 individuals are found in the valley. Therefore, we suggest that the new species *H.stenopterum* should be considered Data Deficient (DD) according to current IUCN Red List Categories and Criteria (IUCN 2022).

## Supplementary Material

XML Treatment for Hiptage stenopterum

## ﻿Additional information

### Author ORCIDs

Tong-Tong Zhang https://orcid.org/0009-0003-9812-2718

Shu-Yun Yang https://orcid.org/0009-0000-8129-5194

Ke Tan https://orcid.org/0000-0002-9036-163X

Ming-Xun Ren https://orcid.org/0000-0002-4707-2656

### Data availability

All of the data that support the findings of this study are available in the main text.
